# The Landscapes of Full-Length Transcripts and Splice Isoforms as Well as Transposons Exonization in the Lepidopteran Model System, *Bombyx mori*

**DOI:** 10.3389/fgene.2021.704162

**Published:** 2021-09-14

**Authors:** Zongrui Dai, Jianyu Ren, Xiaoling Tong, Hai Hu, Kunpeng Lu, Fangyin Dai, Min-Jin Han

**Affiliations:** ^1^ State Key Laboratory of Silkworm Genome Biology, Key Laboratory of Sericultural Biology and Genetic Breeding, Ministry of Agriculture and Rural Affairs, College of Sericulture, Textile and Biomass Science, Southwest University, Chongqing, China; ^2^ WESTA College, Southwest University, Chongqing, China

**Keywords:** full-length transcripts, long noncoding RNA, transposable elements, exonization, *Bombyx mori*

## Abstract

The domesticated silkworm, *Bombyx mori*, is an important model system for the order Lepidoptera. Currently, based on third-generation sequencing, the chromosome-level genome of *Bombyx mori* has been released. However, its transcripts were mainly assembled by using short reads of second-generation sequencing and expressed sequence tags which cannot explain the transcript profile accurately. Here, we used PacBio Iso-Seq technology to investigate the transcripts from 45 developmental stages of *Bombyx mori*. We obtained 25,970 non-redundant high-quality consensus isoforms capturing ∼60% of previous reported RNAs, 15,431 (∼47%) novel transcripts, and identified 7,253 long non-coding RNA (lncRNA) with a large proportion of novel lncRNA (∼56%). In addition, we found that transposable elements (TEs) exonization account for 11,671 (∼45%) transcripts including 5,980 protein-coding transcripts (∼32%) and 5,691 lncRNAs (∼79%). Overall, our results expand the silkworm transcripts and have general implications to understand the interaction between TEs and their host genes. These transcripts resource will promote functional studies of genes and lncRNAs as well as TEs in the silkworm.

## Introduction

As a lepidopteran model organism, the draft genome of *Bombyx mori* has been released 17 years ago (Mita, et al., 2004; Xia, et al., 2004). Subsequently, population genomes and chromosome-level reference genome of the silkworm have been completed one after another (Xia, et al., 2008; Xia, et al., 2009; Xiang, et al., 2018; Kawamoto, et al., 2019). These genome resources play an important role in silkworm domestication history and functional genomics studies (Yang, et al., 2014; Xiang, et al., 2018; Zhu, et al., 2019; Li, et al., 2020; Wang, et al., 2020). Compared with the high-quality genome of the silkworm, the quality of transcripts is poor. The transcripts of *B. mori* so far were mainly assembled by using short reads of second-generation sequencing and expressed sequence tags (ESTs) (Shao, et al., 2012; Suetsugu, et al., 2013).

LncRNAs play important roles in most forms of life (Mercer, et al., 2009; Rinn and Chang 2020). For instance, Locasta migratoria PAHAL lncRNA positively regulates phenylalanine hydroxylase resulting in dopamine production in brain and modulates locust behavioral aggregation (Zhang, et al., 2020). Mouse Braveheart lncRNA contributes to mesoderm and cardiac differentiation (Xue, et al., 2016). Two lncRNAs (roX1 and roX2) of *Drosophila melanogaster* take part in X-chromosome dosage compensation (Ilik, et al., 2013). Human NORAD lncRNA is required for the assembly of topoisomerase complex NARC1, which involve in maintaining genomic stability (Munschauer, et al., 2018). In *B. mori*, systematic characterizations of its lncRNA were studied based on next-generation sequencing (Wu et al., 2016; Zhou et al., 2016; Zhou et al., 2018). Moreover, some studies indicate that some lncRNA may play a role in 20E-induced autophagy (Qiao et al., 2021). However, prior lncRNA based on limited organs and short-read data which cannot fully explain the landscape of lncRNA in silkworms. Additionally, the functions *B. mori* lncRNAs so far remain poorly understood.

Transposable elements (TEs) are the largest component of most eukaryotic genomes, and function in the evolution of genome architecture and gene regulatory network (Feschotte 2008; Kapusta, et al., 2017; Cosby, et al., 2021). Recently, a study in tetrapod showed that a vast majority of transposase DNA binding domains fused to host regulatory domains through exon shuffling (Cosby, et al., 2021). A study in locusts revealed that TEs occupied ∼20% of the locust transcriptome via its exonization (Jiang, et al., 2019). Past studies discovered that ∼40% *B. mori* genome is composed of the known TEs (Osanai-Futahashi, et al., 2008; Xu, et al., 2013). Helitron families of *B. mori* genome contributed to 123 full-length cDNAs (Han, et al., 2013). Nevertheless, in *B. mori*, the contribution of the whole genome TEs to the transcriptome remains unclear.

In this work, we use PacBio Iso-Seq sequencing technology to generate high-quality full-length transcripts from 45 developmental stages of the silkworm. These transcripts are further divided into protein-coding genes and lncRNA based on protein-coding potential and lncRNA characteristics. Finally, the contribution of TEs to the transcripts is investigated.

## Materials and Methods

### Sample Source and RNA Extraction

The silkworm (*Bombyx mori*) strain DaZao in this study was obtained from the Silkworm Gene Bank, Southwest University, China. This strain has been used to generate the reference genome (Kawamoto, et al., 2019). The silkworm was reared on fresh mulberry leaves at 25°C under 12°hours-light/12°hours-dark photoperiod. To obtain as many transcript isoforms as possible, we sampled almost all developmental stages of the silkworm (Supplementary Table S1). Each individual at the larval stage was dissected and then removed food residues in the intestinal to reduce the contamination of mulberry leaves and intestinal microorganisms. Total RNA was extracted using TRIzon Reagent kit No. CW0580S (CoWin Bioscience) and then treated with DNase I (TaKaRa) to remove genomic DNA.

### RNA Library Preparation for SMRT Sequencing

The isolated total RNA (5 μg RNA, equally mixed from each developmental stage sample, Supplementary Figure S1) was used to synthesize cDNA by SMARTer cDNA Synthesis kit (Clontech). Three libraries (1–2 kb, 2–3 kb, and 3–6 kb) were constructed by using Pacific Biosciences DNA Template Prep Kit 2.0. Using the Pacific Bioscience RS II platform, we sequenced 8 SMRT cells including 3 cells for 1–2 kb, 3 cells for 2–3 kb libraries and 2 cells for 3–6 kb libraries.

### RNA Polishing and Non-redundant Transcripts Identification

SMRT Analysis was used to obtain full-length transcripts (v2.3.0, https://www.pacb.com/). Where the polymerase reads with lengths small than 50°bp or quality less than 0.75 were discarded. The full-length reads were defined as containing 5′ and 3′ primers and ployA tail. While the other reads were defined as non-full length reads. The full-length non-chimeric (FLNC) transcripts were defined as full-length ROIs without any additional cDNA sequence. The consensus isoforms were obtained by using ICE (Iterative Clustering for Error Correction) with default parameters. The consensus isoforms were polished using Quiver (parameters: -hq_quiver_min_accuracy 0.99). High-quality consensus isoforms were classified with the criteria post-correction accuracy above 99%. Then the high-quality consensus isoforms were mapped to the reference genome using GMAP (Wu and Watanabe, 2005) (2017–11–15, parameters: -direction sense_force--cross_species--allow_close_indels 0). The sequences with identity less than 0.9 or coverage less than 0.85 were filtered using the pbtranscript-ToFU package (parameters: -min-trimmed-coverage = 0.85°min-identity = 0.9). The non-redundant high-quality consensus isoforms were obtained by merging sequences that differ only at the 5’ terminal exon and the other exons were identical.

### Alternative Splicing Analysis and Verification

Alternative splicing (AS) events including Intron Retention (IR), Exon skipping (ES), Alternative 5′ splice site (A5S), Alternative 3’ splice site (A3S), and Mutually exclusive exon (MEE) were identified by the AStalavista tool (Foissac and Sammeth 2007). To validate alternative splicing events, five AS events were randomly selected to perform RT-PCR and sanger sequencing. The gene-specific primer was designed based on flanking regions of each splicing site using NCBI primer-Blast tool (https://www.ncbi.nlm.nih.gov/tools/primer-blast/). The five pairs of primers were listed in Supplementary Table S2.

### Long Non-Coding RNA Identification, Open Reading Frames Identification, and Functional Annotation

All non-redundant transcripts were used to identify Long non-coding RNA (lncRNAs) and open reading frames (ORFs). The LncRNAs were identified by using Coding Potential Calculator (CPC2) (Kang, et al., 2017), Coding-Non-Coding Index (CNCI) (Sun, et al., 2013), Coding-Potential Assessment Tool (CPAT) (Wang, et al., 2013), and an ab initio lncRNA identification tool (LncAdeep) (Yang, et al., 2018). The ORFs were identified by using TransDecoder software (http://transdecoder.sourceforge.net/). All ORFs were annotated by WEGO (Ye, et al., 2018), an online gene ontology website (http://wego.genomics.cn/), and based on COG (clusters of Orthologous Groups) database (Tatusov, et al., 2000).

### Identification of Transcripts With Transposable Elements Exonization

The silkworm repeat library (Xu, et al., 2013) was used to identify transposon sequences in all non-redundant transcripts by RepeatMasker v4.1.0 (http://repeatmasker.org/) with RMBlast v2.9.0 (http://www.repeatmasker.org/RMBlast.html).

## Results

### Transcriptome Sequencing Using SMRT

To obtain more transcripts of *Bombyx mori*, the total RNAs of whole-body samples from 45 developmental stages were extracted for long-reads sequencing (PacBio RS II platform) (Supplementary Figure S1). We obtained 777,701 polymerase reads and 7,100,417 subreads (15.88Gb clean data) by PacBio sequencing (Supplementary Table S3). Based on the number of full passes >0 and accuracy >0.75, a total of 516,326 reads of insert were generated (Supplementary Table S4). The average length of ROI from 1 to 2kb, 2–3kb, and 3–6kb cDNA size library were 1,736bp, 2,628bp, and 3,819bp, respectively (Supplementary Table S4). The observed distribution of ROI length for each cDNA size library was consistent with the expected (Supplementary Figure S2A-C). After filtering short-length ROIs (<300bp), there were 286,153 full-length ROI (containing 5′primer, 3′ primer, and polyA tail) and 196,776 non-full-length ROI (Supplementary Table S5). After filtering chimeric ROIs, 285,496 ROIs were identified as full-length non-chimeric (FLNC) reads constituted 55.3% of all ROIs (Supplementary Figure S3A). The distribution of FLNC length for each cDNA size library was shown in Supplementary Figure S2D–E. The average length of FLNC reads from 1 to 2kb, 2–3kb, and 3–6kb cDNA size library were 1,340bp, 2,364bp, and 3620bp, respectively (Supplementary Table S5).

### Isoforms Clustering, Error Correction, and Alternative Splicing Analysis

**FIGURE 1:**
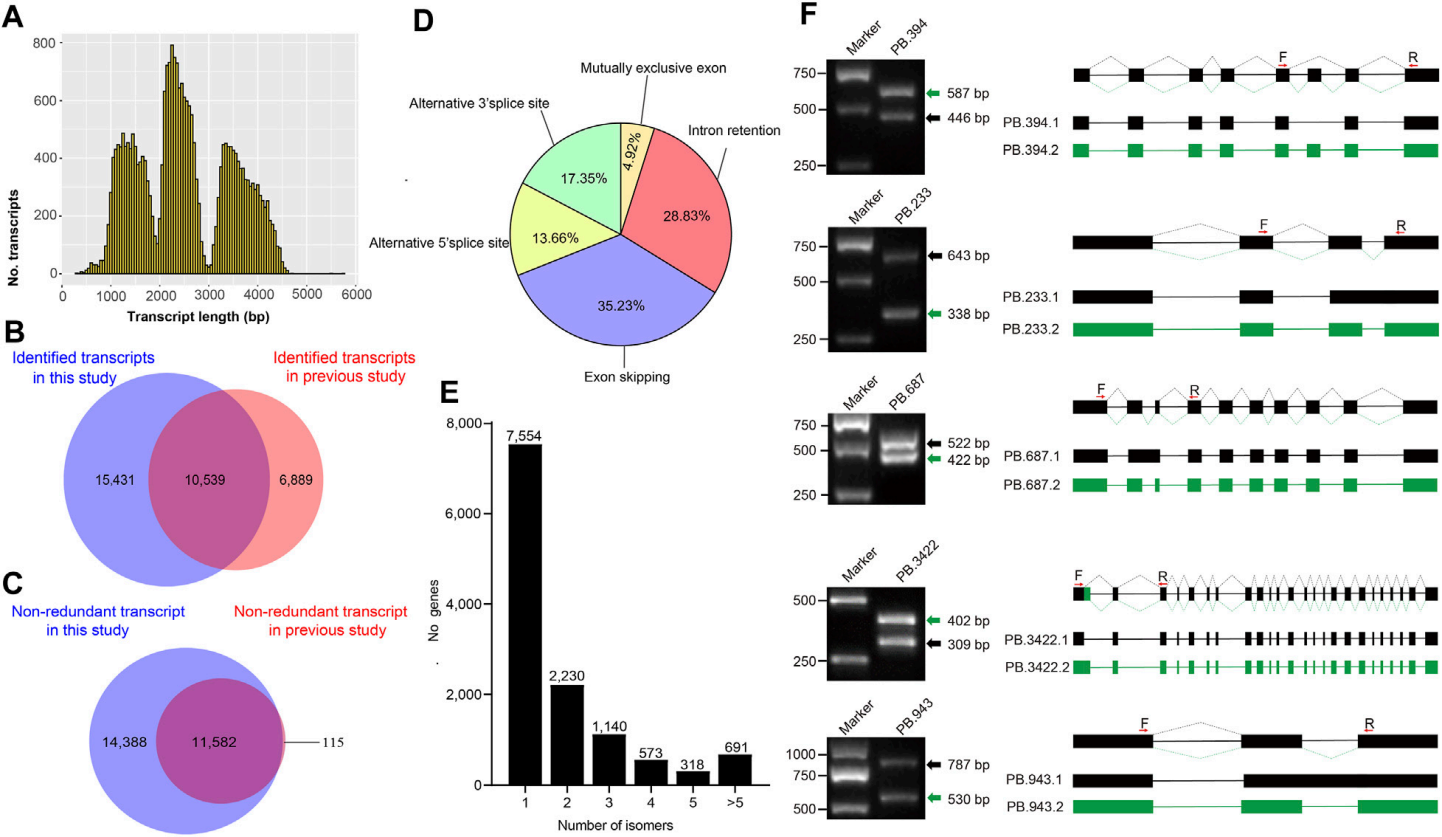
Non-redundant transcripts and alternative splicing events (**A**) The distribution of all non-redundant transcripts length (**B**) The Venn diagram shows the comparative results of the transcript identified in this study and the previously reported transcript (**C**) The Venn diagram shows the comparative result between the non-redundant transcripts identified in this study and the previously reported transcripts in silkworm silk gland (**D**) Proportion of each alternative splicing type (**E**) Distribution of splice isoforms of genes (**F**) The random selected alternative splicing events were verified by Polymerase Chain Reaction (PCR) and Sanger sequencing. Primers (F represent forward primer and R represent reverse primer) were designed based on flanking regions of alternative splicing sites. The PCR product was run on 1% agarose gel and observed in UV light. For each gel, the green and black arrows indicate different splicing isoforms. The structures of the isoforms are showed on the right side of the figure. The filled boxes represent Exons and lines represent introns. Different isoforms are showed with different color box connected by lines. The red arrows upon the boxes represent the locations sites of the primers.

To obtain consensus isoforms, all ROIs were clustered by iterative isoform-clustering (ICE) algorithm. We obtained 69,427 ICE consensus isoforms. Among these isoforms, the number of isoforms from the sequence length of <1kb, 1–2kb, 2–3kb, 3–6kb, and >6kb were 2,249, 24,180, 26,438, 15,913, and 647, respectively (Supplementary Table S6 and Supplementary Figure S3B–F). To filter low-quality isoforms, the consistent sequences of each cluster were corrected and evaluated by quiver program. We obtained 53,508 high-quality isoforms (HQs) with accuracy >99% and 15,919 low-quality isoforms (LQs) (Supplementary Table S6). The HQs constitute 25,970 non-redundant high-quality consensus isoforms (nrHQCIs) by merging high-quality isoforms that differ only at the 5’ terminal exon and the other exons were identical (Supplementary Table S7). The distribution of nrHQCIs length was consistent with the expected size of the three libraries (Figure 1A). The average length of nrHQCIs was 2,504 bp, and there were 25,332 nrHQCIs (∼97.5%) with sequence length of more than 1,000 bp. Compared with the previous transcripts assembled by short reads of second-generation sequencing and expressed sequence stags, 15,431 transcripts out of 25,970 nrHQCIs were novel (Figure 1B and Supplementary Table S8). Meanwhile, 6,889 transcripts were found in previous studies but not in this study. This phenomenon could be caused by that our sequencing depth was not enough, and some low-expressed genes may not be detected. We further compared our nrHQCIs with prior identified 11,697 nrHQCIs based on silk gland long-read transcriptome (Chen et al., 2020), 11,582 (∼91%) 11,697 or prior nrHQCIs were overlapped in our 25,970 nrHQCIs (Figure 1C) and there were 14,388 new transcripts in our nrHQCIs.

To detect alternative splicing (AS) events, the AStalavista tool was used to identify Intron Retention (IR), Exon skipping (ES), Alternative 5′ splice site (A5S), Alternative 3′ splice site (A3S), and mutually exclusive exon (MEE) events. We detected a total of 18,416 AS events, and the majority of AS events being Exon skipping (Figure 1D). The distribution of isoform numbers of genes was shown in Figure 1E. Only one isoform was detected for 7,554 genes, and 4,952 genes produced two or more transcripts. To validate the accuracy of the detected AS events, five genes were randomly selected to perform RT-PCR and Sanger sequencing. The size of the gel band and the results of Sanger sequencing were consistent with the detected AS isoforms (Figure 1F).

**FIGURE 2:**
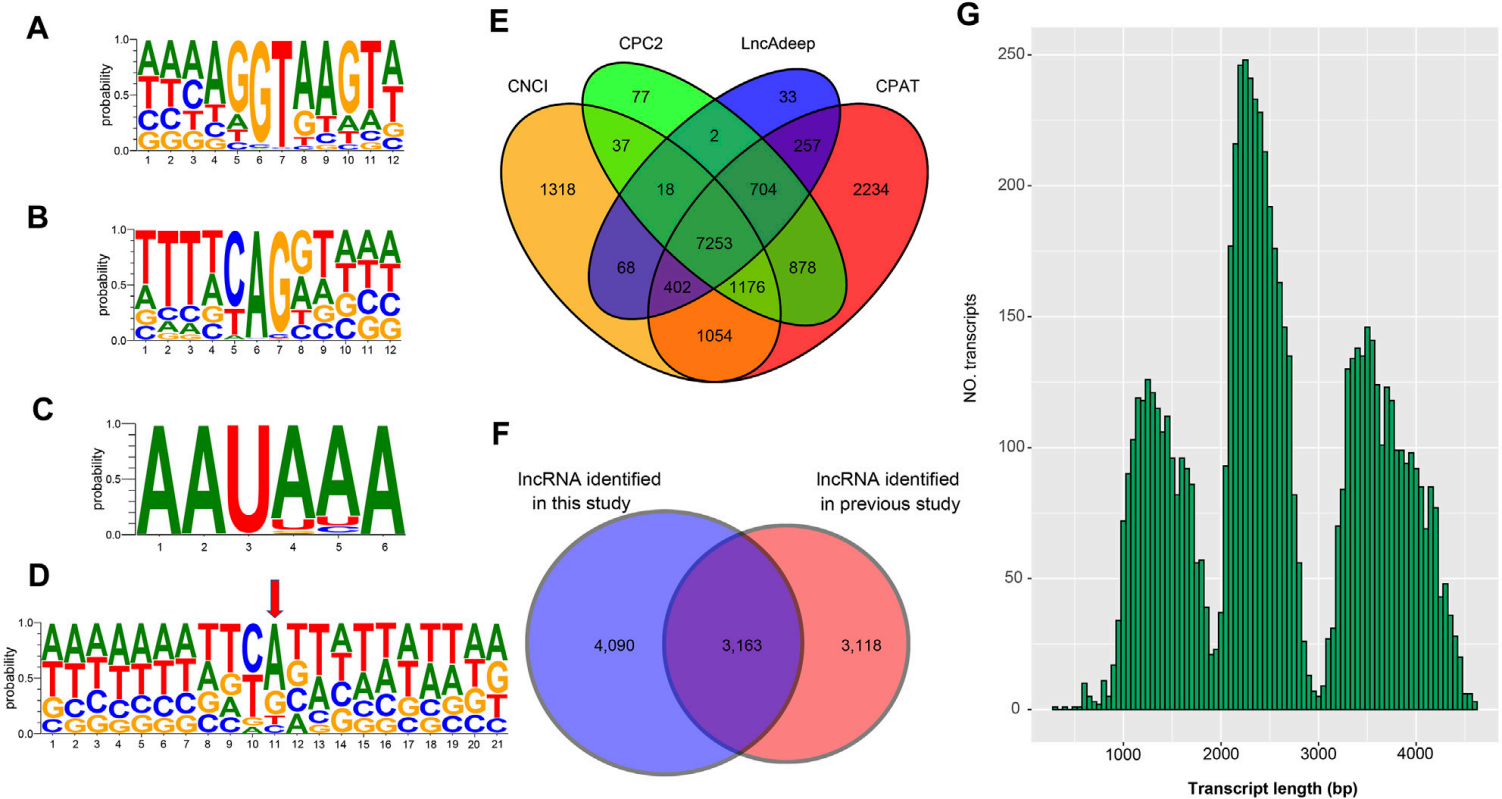
Analysis of splice junctions, poly(A) signal sites, TSS site, and lncRNA (**A**) The mononucleotide variant distributions of the donor site (**B**) The mononucleotide variant distributions of the acceptor sites (**C**) The mononucleotide variant distributions of the poly(A) site (**D**) The mononucleotide variant distributions of the TSS sites **(E)** Venn diagram of lncRNA identification by four methods including CNCI, CPC2, LncAdeep and CPAT (**F**) The Venn diagram shows the comparative result of the lncRNA identified in this study and the previously reported LncRNAs (**G**) The length distribution of the lncRNAs identified in this study.

To verify the 25,970 nrHQCIs, splicing sites, TSS sites, and AS events were verified by next-generation sequencing (NGS) data. The results revealed that 105,468 (91%) out of 116,399 splicing sites, 16,021 (∼87%) of 18,416 AS events of 25,970 nrHQCIs were verified by NGS data. However, only 8,851 (∼34%) transcription start sites (TSSs) were verified by NGS data (Supplementary Table S8). This phenomenon may be caused by the construction method of the PacBio library that did not take into account the 5′CAP integrity of RNA. Besides, the TSS sites identified by NGS data may not be accurate. Furthermore, we analyzed the consensus motif around donor and acceptor sites, polyadenylation sites, and (TSS). We found that the splicing donor and acceptor sites have conserved GT-AG consensus motif (Figures 2A,B), which is consistent with the other organism such as perennial ryegrass (Xie, et al., 2020), *B. malayi* (Nicolas J Wheeler, et al., 2020), and Medicago sativa L. (Chao, et al., 2019). In addition, the result of polyadenylation sites analysis indicates that “AAUAAA” has the highest frequency (Figure 2C). This result has highly corresponded with other studies (Wheeler, et al., 2020; Zhao et al., 2019).

### Long Non-Coding RNA Identification and Open Reading Frames Prediction as Well as Protein-Coding Genes Annotation

To detect lncRNA in the 25,970 nrHQCIs, we used CNCI, CPC2, CPAT, and LncAdeep programs to identify lncRNAs. We took RNAs that all four software considered as lncRNAs as lncRNAs. A total of 7,253 nrHQCIs were detected as lncRNAs (Figure 2E). Compared with the previously reported lncRNAs identified by next-generation high-throughput sequencing technology (NGST), 4,090 lncRNA were novel (Figure 2F and Supplementary Table S9). The average length of identified lncRNAs was 2,585 bp, and the length distribution of lncRNAs was showed in Figure 2G.

**FIGURE 3:**
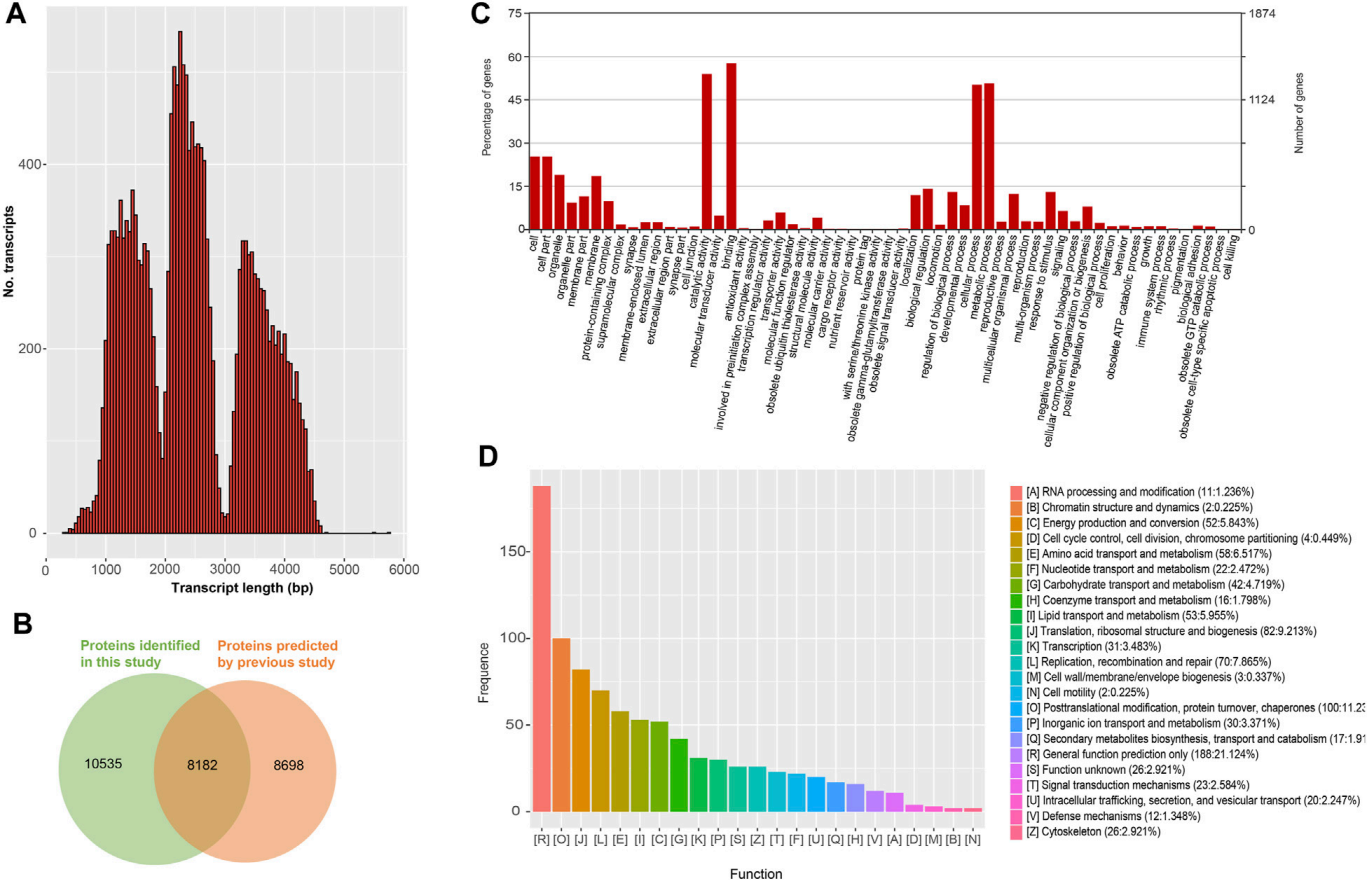
Protein coding transcripts **(A)** The distribution of all protein coding transcripts length (**B**) Venn diagram of the protein coding transcripts identified in this study and previously reported protein coding transcripts (**C**) Gene Ontology (GO) terms distribution of all protein coding transcripts (**D**) Clusters of Orthologous Groups (COGs) of all protein coding transcripts.

The length distribution of the remained 18,717 nrHQCIs was showed in Figure 3A, and the protein-coding sequences were predicted by TransDecoder. Compared with the previously predicted protein, 10,535 predicted proteins were novel (Figure 3B and Supplementary Table S10). The 18,717 nrHQCIs were functionally annotated by searching the databases of COG, GO, KEGG, KOG, Pfam, Swissprot, eggnog, and NR. A total of 9,942 nrHQCIs were annotated (Supplementary Table S11). For instance, 5,569 nrHQCIs were annotated by GO analysis (Figure 3C). For COG annotation, 2,111 nrHQCIs were annotated, and the largest category was “General function prediction only” (Figure 3D). This finding is consistent with the observation in Medicago sativa L (Chao, et al., 2019).

### Transposon Exonization

**FIGURE 4:**
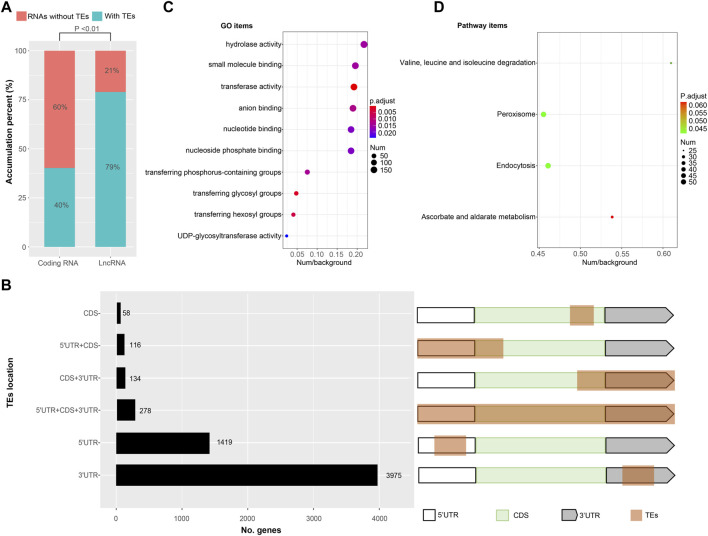
Transcripts with transposable elements (TEs) exonization **(A)** The proportion of TEs exonization transcripts in protein coding transcripts and lncRNAs, respectively (**B**) The location distribution of TEs derived sequences in protein coding transcripts. White boxes represent 5′UTR, green boxes represent coding DNA sequences (CDS), gray boxes represent 3′UTR and brown box represent TEs derived sequences (**C**) GO enrichment results of TEs exonization transcripts (**D**) KEGG enrichment results of TEs exonization transcripts.

Transposable elements (TEs) constitute a significant component (∼40%) of the *Bombyx mori* genome (Osanai-Futahashi, et al., 2008; Xu, et al., 2013). However, TEs contribute to the transcripts of *Bombyx mori* remain unclear. Here, transcripts with TEs were identified by homology searching. We found the number of transcripts with TEs was 11,671 (∼45% of all identified nrHQCIs) containing 5,980 (∼32% of all protein-coding transcripts) protein-coding transcripts and 5,691 (∼79% of all identified lncRNAs) lncRNAs (Figure 4A). The proportion of TEs exonization in lncRNA was significantly higher (two-sample test of proportions, *p* < 0.01) than the proportion of TE exonization in the protein-coding transcripts (Figure 4A). For 5,980 protein-coding transcripts with TEs, there were 278 transposase coding transcripts, and the remain 5,702 protein-coding transcripts composed of TEs fragments plus other non-transposase coding genes (Figure 4B). Where TEs contributed to the 5′UTRs of 1,535 protein-coding transcripts discarded transposase transcripts, to the 3′UTRs of 4,109, and to the ORFs of 308 (Figure 4B and Supplementary Table S12).

For 5,702 protein-coding transcripts with TEs, GO enrichment analysis showed that these genes enriched in 10 GO items including hydrolase activity, small molecule binding, transferase activity, anion binding, nucleotide binding, nucleoside phosphate binding, transferring phosphorus-containing groups, transferring glycosyl groups, transferring hexosyl groups and UDP-glycosyltransferase activity (Figure 4C and Supplementary Figure S5). KEGG enrichment analysis showed that the genes enriched in four pathway items including valine/leucine/isoleucine degradation, peroxisome, endocytosis, and ascorbate/alternate metabolism (Figure 4D).

## Discussion

*B.mori* was an important lepidopteran model system. Although the high-quality reference genome has been released in 2019 (Kawamoto et al., 2019), the transcriptome so far was obtained through assembling short reads resulting in poor-quality transcripts and incorrect genome annotation (Shao et al., 2012; Suetsugu et al., 2013). Recently, with the development of sequencing technology, PacBio sequencing, which has a profound advantage in long reads, has been applied widely to generate high-quality full-length transcripts in eukaryotes (Sharon et al., 2013; Wang et al., 2016; Jiang et al., 2019; Wang et al., 2019; Yang et al., 2020; Xu et al., 2021). In this study, we identified 25,970 high-quality transcripts in the silkworm by using PacBio sequencing technology. Compared with prior identified full-length transcripts based on EST and silk gland long-read transcriptom (Chen et al., 2020; Suetsugu et al., 2013), we identified 15,431 and 14,388 new transcripts, respectively, which will improve the genome annotation, and promote functional genomic studies.

*B.mori* is the only truly domesticated insect. Long-term artificial selection has resulted in significant differences between the domesticated silkworm and its ancestors (Bombyx mandarina) in traits such as silk yield, behavior, body color and so on (Li, et al., 2020; Lu, et al., 2020; Wang, et al., 2020). Besides, more than 3,000 silkworm strains and >600 mutations with diverse phenotypes are available worldwide which are generated through spontaneous mutation, artificial mutagenesis, or breeding (Nagaraju et al., 2000; Goldsmith, et al., 2005; Furdui, et al., 2014). As far as we know, more than 60 mutations so far have been deciphered. For instance, the twin-spot markings on *B. mori* larval are caused by periodic Wnt1 expression (Yamaguchi, et al., 2013). The Toll ligand Spz-3 controls the black stripe of each segment of the silkworm (Kondo, et al., 2017). The BmGlcNase1 gene is involved in the synthesis of sericin (Li, et al., 2020). However, a great number of control genes of the traits of domestication and mutation remain unclear. The high-quality reference transcriptome of the silkworm obtained in this study will facilitate the deciphering of these traits.

LncRNAs are widespread in eukaryotes and play an important role in gene-regulatory networks (Kopp and Mendell, 2018). Prior studies of *B. mori* identified 6,281 possibly lncRNAs through second-generation sequencing technology and found two lncRNAs that could be related to silk protein translation (Zhou et al., 2016; Zhou et al., 2018). However, the function of the vast majority of *B. mori* lncRNAs remains unknown. Moreover, these lncRNAs maybe not accurate due to the limitation of short reads. Here, we identified 7,253 high-quality lncRNAs with 4,090 completely novel lncRNAs through long-read sequencing technology. The studies of the function and biological relevance of these lncRNAs are interesting topics in the future.

Transposable elements (TEs) are mobile elements and are powerful mutagens that play important roles in eukaryotic genome evolution and adaptation as well as disease (Lisch 2013; Chuong et al., 2017; Payer and Burns, 2019). TEs occupied ∼40% of the *B. mori* genome (Osanai-Futahashi et al., 2008; Xu et al., 2013). Moreover, past studies revealed that a large number of traits of domestication and mutation are caused by TEs transposition. For example, the trait of developmental uniformity of *B. mori* is attributed to a non-LTR transposon (Taguchi) inserted in upstream of the silkworm ecdysone oxidase (Sun et al., 2014). A Tc1-mariner transposon inserted in the upstream of tyrosine hydroxylase is responsible for the sex-linked chocolate (sch) mutant of *B. mori* (Liu et al., 2010). A transposon-associated genomic deletion is involved in the trait of white cocoon (Sakudoh et al., 2007). Howbeit, the mechanism of the large majority of silkworm traits are unknown. In this work, we identified 11,671 transcripts with TEs exonization. Which has general implication for understanding the evolution of genes. Furthermore, whether transposon can alter gene structure, function or expression through its exonization to control the trait of *B. mori* is another interesting question in future.

## Data Availability

The PacBio sequencing data has already been submitted to CNGBdb (China National GeneBank DataBase. The sequencing ID is CNP0001781. The information of experimental samples is accessible with the sample ID CNS0360521). Details for the project can be searched through the project ID CNP0=001781. The SRA data has also been released in NCBI (National Center for Biotechnology Information). The Biosample and Bioproject ID are SAMN20348872 and PRJNA748960.
