# Supplementary material for: The Landscapes of Full-Length Transcripts and Splice Isoforms as Well as Transposons Exonization in the Lepidopteran Model System, Bombyx mori
Source: Front Genet. 2021 Sep 14;12:704162. doi: 10.3389/fgene.2021.704162 (PMC8476886; doi:10.3389/fgene.2021.704162)
Supplement: Supplementary file 4 [file Table1.DOCX]

**Supplementary table 1: The 45 sampling in each developmental stages of silkworm**

| Stages | Sampling number |
| --- | --- |
| Egg | 19 |
| Larva | 17 |
| Spinning cocoons | 2 |
| Pupa | 5 |
| Moth | 2 |
| Total | 45 |
