# Supplementary figures and images for: The Landscapes of Full-Length Transcripts and Splice Isoforms as Well as Transposons Exonization in the Lepidopteran Model System, Bombyx mori
Source: Front Genet. 2021 Sep 14;12:704162. doi: 10.3389/fgene.2021.704162 (PMC8476886; doi:10.3389/fgene.2021.704162)

Supplementary fig. 3

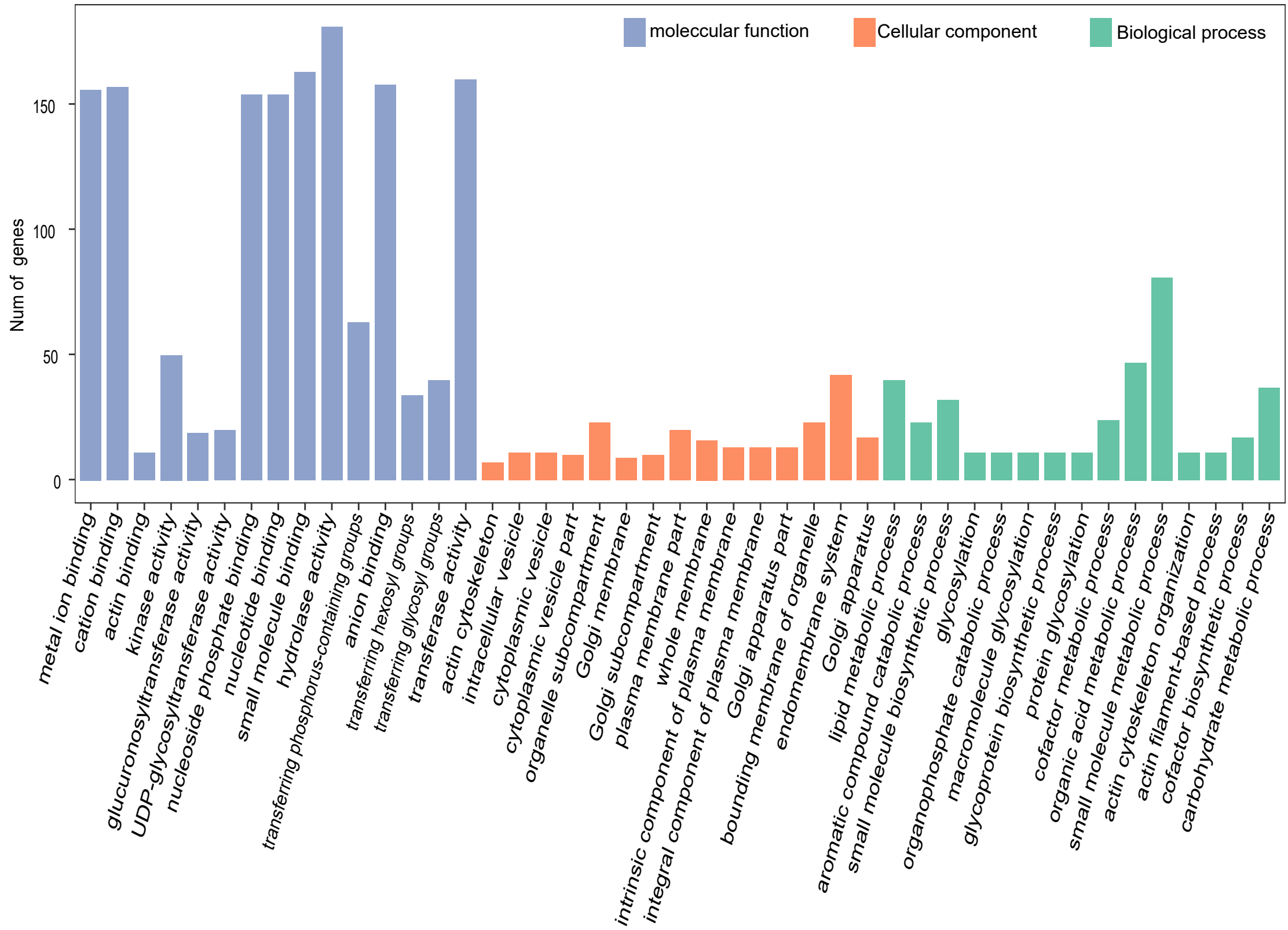

Supplement: Supplementary file 6 [file DataSheet3.PDF]

**Supplementary fig.1**

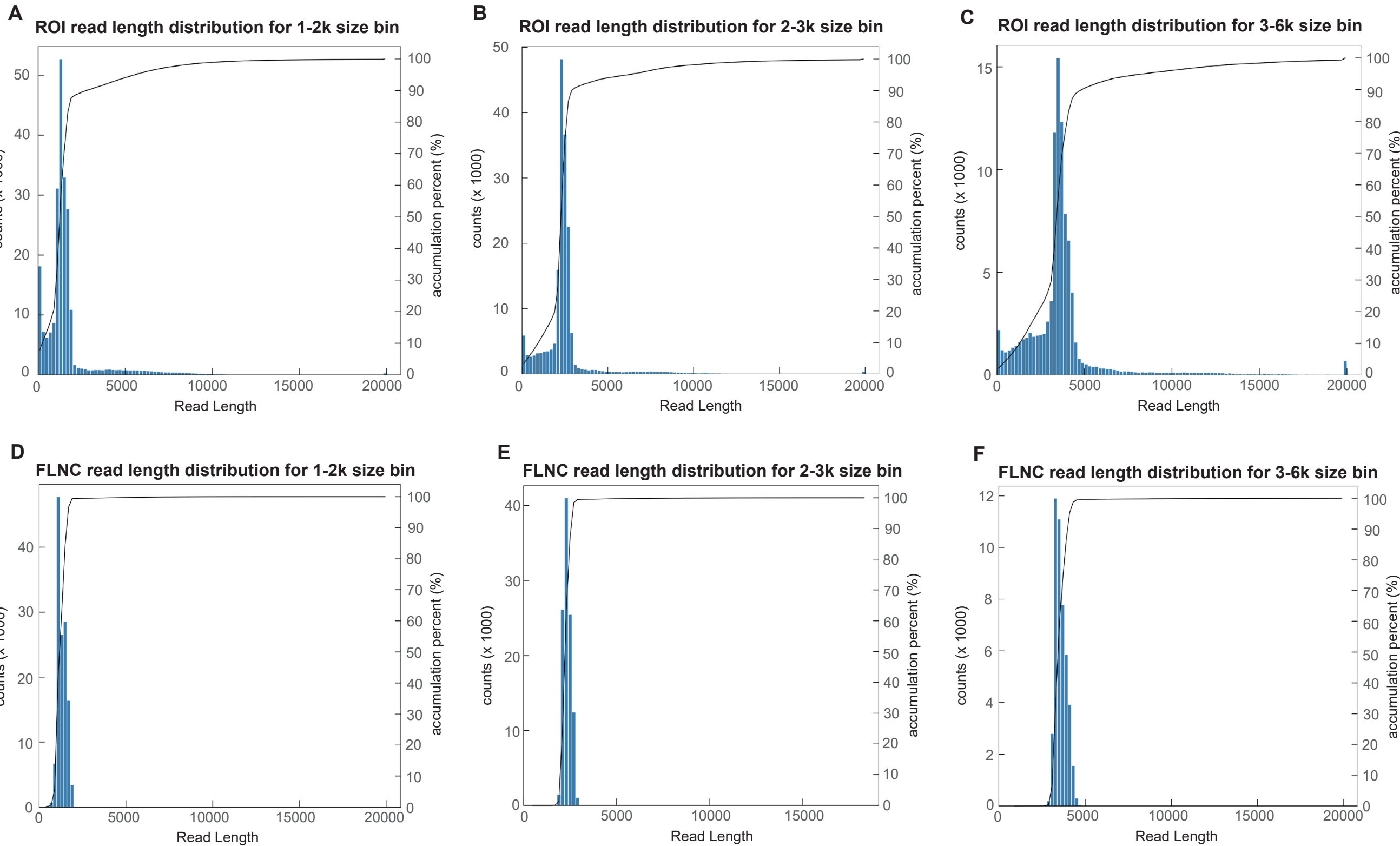

Supplement: Supplementary file 7 [file DataSheet1.PDF]
