# Supplementary material for: The Landscapes of Full-Length Transcripts and Splice Isoforms as Well as Transposons Exonization in the Lepidopteran Model System, Bombyx mori
Source: Front Genet. 2021 Sep 14;12:704162. doi: 10.3389/fgene.2021.704162 (PMC8476886; doi:10.3389/fgene.2021.704162)
Supplement: Supplementary file 8 [file Table4.DOCX]

**Supplementary table 4** Summary of ROIs for PacBio sequencing

| **cDNA size** | **Reads of Insert** | **Read Bases of Insert** | **Mean Read Length of Insert** | **Mean Read Quality of Insert** | **Mean Number of Passes** |
| --- | --- | --- | --- | --- | --- |
| 1-2K | 231,371 | 401,889,401 | 1,736 | 0.94 | 13.00 |
| 2-3K | 184,150 | 484,092,841 | 2,628 | 0.92 | 8.00 |
| 3-6K | 100,805 | 385,034,716 | 3,819 | 0.88 | 5.00 |
