# Supplementary material for: The Landscapes of Full-Length Transcripts and Splice Isoforms as Well as Transposons Exonization in the Lepidopteran Model System, Bombyx mori
Source: Front Genet. 2021 Sep 14;12:704162. doi: 10.3389/fgene.2021.704162 (PMC8476886; doi:10.3389/fgene.2021.704162)
Supplement: Supplementary file 9 [file Table11.DOCX]

**Supplementary table 11 Transcripts annotated by searching public databases**

| **Annotated database** | **Annotated Number** | **Percentage(%)** |
| --- | --- | --- |
| COG Annotation | 2111 | 11.27 |
| GO Annotation | 5596 | 29.90 |
| KEGG Annotation | 3842 | 20.53 |
| KOG Annotation | 5337 | 28.51 |
| Pfam Annotation | 5554 | 29.67 |
| Swissprot Annotation | 5318 | 28.41 |
| eggnog Annotation | 9257 | 49.46 |
| Nr Annotation | 9731 | 51.99 |
| All Annotated | 9942 | 53.12 |
| All Analysed | 18717 | 100 |
