# Supplementary material for: The Landscapes of Full-Length Transcripts and Splice Isoforms as Well as Transposons Exonization in the Lepidopteran Model System, Bombyx mori
Source: Front Genet. 2021 Sep 14;12:704162. doi: 10.3389/fgene.2021.704162 (PMC8476886; doi:10.3389/fgene.2021.704162)
Supplement: Supplementary file 10 [file Table6.DOCX]

**Supplementary table 6** Summary of consensus isoforms for the PacBio sequencing.

| **Size (kb)** | **Number of consensus isoforms** | **Average length of consensus isoforms** | **Number of polished high-quality isoforms** | **Number of polished low-quality isoforms** | **Percent of polished high-quality isoforms(%)** |
| --- | --- | --- | --- | --- | --- |
| 0-1 | 2,249 | 883 | 2,098 | 151 | 93.29% |
| 1-2 | 24,180 | 1,410 | 20,847 | 3,333 | 86.22% |
| 2-3 | 26,438 | 2,379 | 20,846 | 5,592 | 78.85% |
| 3-6 | 15,913 | 3,677 | 9,704 | 6,209 | 60.98% |
| >6 | 647 | 8,978 | 13 | 634 | 2.01% |
